# Unclear boundaries: Family caregivers’ invisible role in home-based palliative care in Norway: an exploratory analysis of secondary data from two previous studies

**DOI:** 10.1016/j.ijnsa.2026.100559

**Published:** 2026-05-15

**Authors:** Anett Skorpen Tarberg, Bodil Aarmo Brenne, Kristin Jeppestøl, Bente Egge Søvde, Katrine Staats

**Affiliations:** a Department of Health Sciences in Ålesund, Faculty of Medicine and Health Sciences, NTNU, Larsgårdsvegen 2, Postboks 1517, 6025, Ålesund, Norway; b Nord University, Faculty of Nursing and Health Sciences, Høgskoleveien 27, 7600, Levanger, Norway; c University of Agder, Faculty of Health and Sport Sciences, Department of Health and Nursing Science, Jon Lilletunsvei 9, 4879, Grimstad, Norway; d Department of Health and Caring Sciences, Western Norway University of Applied Sciences, Svanehaugsvegen 1, 6800, Forde, Norway; e OsloMet – Oslo Metropolitan University, Faculty of Health Sciences, Department of Nursing and Health Promotion, Head of Studies - Area of responsibility 2, Kunnskapsveien 55, 2007, Kjeller, Norway

**Keywords:** Family caregivers, Palliative care, Primary healthcare, Home care services, Neoplasms, Health literacy

## Abstract

**Background:**

The future need for home-based palliative care is expected to increase significantly. Family caregivers play a crucial role in home settings by ensuring continuity of care, participating in decision-making, and providing both emotional and practical support to their family members. Despite guidelines highlighting the importance of their involvement in providing palliative care at home, inadequate support from healthcare professionals often leaves family caregivers feeling overwhelmed and undervalued.

**Objective:**

This study aimed to explore the pivotal role of family caregivers in home-based palliative care to deepen our understanding of their experiences and challenges as they navigate their undefined roles between healthcare professionals and patients.

**Design:**

This study was a qualitative and explorative secondary analysis of data from two prior studies, guided by Gadamer’s hermeneutical approach.

**Settings:**

All interviews took place in the participants’ homes, except for two in public institutions and two at work locations.

**Participants:**

Twenty-four male and female family caregivers (age range, 30–80 years).

**Methods:**

Using purposive sampling, data were collected through individual interviews with family caregivers. Oncology nurses and cancer co-ordinators facilitated the recruitment of participants. Secondary data analyses were conducted using Gadamer’s hermeneutical circle.

**Results:**

Three main themes were identified. First, *being lost in an unclear palliative care pathway* where the family caregivers described themselves as “pawns” with an overwhelming burden. Second, *feeling invisible in the caregiving role* reflected how many family caregivers felt unnoticed despite their crucial contribution to patient care. Their well-being and needs were rarely addressed, leaving them struggling with their own emotions. Third, *being assigned unclear responsibilities and tasks beyond capacity*. These family caregivers felt overwhelmed by their responsibility for their patients, compounded by insufficient information and dialogue, leaving them uncertain about what to expect. Interpreting their patients’ condition without competent assistance led to feelings of insecurity and unpredictability.

**Conclusions:**

To support family caregivers adequately in their ambiguous role within palliative care, healthcare professionals must foster open dialogue, ensure clear communication, and provide consistent support. Family caregivers often face emotional and practical challenges; therefore, their often invisible contributions should be acknowledged through increased involvement in care planning, structured emotional support, and formal recognition. Systematic approaches with transparent care pathways offering guidance to enable family caregivers to understand and manage their perceived responsibilities must be established. Hence, recognizing the significance of family caregivers’ roles and enhancing their health literacy are critical to improving the well-being of both patients and their families.

**What is already known**

• Family caregivers play a critical yet often invisible role in home-based palliative care by providing emotional and practical support to their family members
• Healthcare professionals often provide insufficient support for family caregivers, leading to undue emotional and psychological stress and burdens.

Alt-text: Unlabelled box dummy alt text

**What this paper adds**

• In supporting family caregivers, it is essential for healthcare professionals to consistently foster open dialogue and ensure clear communication to help them navigate the emotional and practical challenges they face in home-based palliative care.
• Transparent care pathways with clear guidance from healthcare professionals enable family caregivers to understand and manage their self-perceived and unclear responsibilities.
• Recognizing the significant role of family caregivers and strengthening their collaboration with healthcare professionals are critical to promoting well-being among patients and their families, which reduces family caregivers’ psychological stress.

Alt-text: Unlabelled box dummy alt text

## 1 Background

By 2050, the global population aged 65 and older is estimated to exceed 1.5 billion (Stibbe et al., 2020). This demographic shift will significantly increase the number of home-dwelling older adults with age-related chronic conditions. In response, policymakers and stakeholders advocate enabling patients to stay at home longer and die in familiar surroundings, which aligns with many patients’ end-of-life preferences (Fereidouni et al., 2021; Ministry of Health and Care Services, 2020b; Staats et al., 2023). Early initiation of palliative care is also recommended because it enhances treatment efficacy, reduces symptom crises, and prevents information gaps (Allen et al., 2022; Bradley et al., 2025; Verkissen et al., 2019). Palliative care improves patients’ and families’ quality of life satisfaction, reduces hospital deaths, and minimizes the need for overly aggressive end-of-life interventions for individuals experiencing life-threatening illnesses (Hui et al., 2018; Radbruch et al., 2020). Consequently, the demand for both social and professional support systems skilled in delivering home-based palliative care is growing (Hov et al., 2020; Schutter et al., 2025), which not only includes medical expertise but also holistic approaches that address essential practical and emotional needs for both patient care and supporting informal caregivers (De Boer et al., 2017; Engel et al., 2023; Staats et al., 2025).

Family caregivers (FCs) play a pivotal role in home-based palliative care by providing care, ensuring continuity, and actively making decisions on behalf of critically ill family members, which highlights their integral role in the healthcare system (Bijnsdorp et al., 2022; Hov et al., 2020). However, this responsibility can be overwhelming (Mohammed et al., 2018). In particular, it is important for healthcare professionals to involve FCs in treatment planning and provide emotional and social support without pressuring them to assume home caregiving roles (Woodman et al., 2016; Adejoh et al., 2021). Unfortunately, healthcare professionals often provide insufficient support to FCs, which causes undue stress and burden (McDonald et al., 2018; Naoki et al., 2018; Matthys et al., 2022). Despite legal rights (Norwegian Ministery of Health and Care Service-Patients and User Rights, 1999), national guidelines (Norwegian Directorate of Health, 2017), and supportive policies (Ministry of Health and Care Services, 2020b), Norwegian FCs are inadequately involved in the care of family members nearing their end of life (Symmons et al., 2023). Hence, FCs’ contributions to palliative care are undervalued, and opportunities to provide healthcare knowledge to enhance patient safety are lacking. The Norwegian National Health and Co-ordination Plan 2024–2027 explicitly emphasizes the need to strengthen FC involvement at all levels of palliative care (Ministry of Health and Care Services, 2024). However, balancing caregiving burdens with emotional rewards is still a complex challenge. For many FCs, this balance depends on whether they perceive their home as the most suitable place for their family members to die. Any discordance in care preferences between patients and their FCs often arises from limited mutual awareness and communication; therefore, open dialogues and advanced care planning can help foster consensus (Symmons et al., 2023). While patients may retain a sense of normalcy at home, FCs often experience the opposite (Pottle et al., 2017). Healthcare professionals sometimes fail to recognize this contrast, and inadequate involvement of FCs in decision-making practices can cause dissatisfaction and stress (Schulz et al., 2020; Naoki et al., 2018). This underscores the critical importance of providing FCs with comprehensive information and fostering transparent communication to help them navigate the complex landscape of home-based palliative care (Røen et al., 2018; den Herder-van der Eerden et al., 2017).

Therefore, *health literacy* is a crucial component of the successful inclusion of FCs in home-based palliative care. Health literacy is defined as the ability to access, understand, and use health-related information and services (Liu et al., 2020). As a critical component of the FCs’ toolkit for home-based palliative care, health literacy equips FCs with the knowledge and skills needed to navigate the healthcare system, interpret medical instructions, and actively participate in informed decision-making regarding care for their family member (Papadakos et al., 2022; Yuen et al., 2018, 2024). Against this backdrop, this study explores the pivotal role of FCs in home-based palliative care and deepens our understanding of their experiences and challenges as they navigate their undefined roles between healthcare professionals and patients. Hence, this study examines how FCs experience role ambiguity in their interactions with healthcare professionals supporting home-based palliative care services.

## 2 Methodology

This study employs a qualitative, explorative, and descriptive design based on data from two previously completed studies, which were independently conducted by the first and last authors in 2017 and 2019, respectively. We will refer to these primary studies in our secondary analysis, which involves 24 FCs (Table 1), and explore new research questions using previously gathered data (Heaton, 2004; Long-Sutehall et al., 2011). Secondary analyses are especially important when dealing with a target group in vulnerable life situations that are difficult to reach (Long-Sutehall et al., 2011). Researchers are obliged to carefully consider the ethical implications and strive to minimize individuals’ burdens and limit their participation frequency in research (Conway et al., 2023). Analytical methods that align with those of the primary research are recommended; therefore, we used Gadamer’s hermeneutical methodology to interpret the transcribed texts, which emphasizes the value of the hermeneutic circle (Gadamer, 2010). Consequently, we were able to gain a deeper understanding of the meaning of the texts, continually revising and adjusting our preunderstandings.

**Table 1:** Characteristics of study participants.

	Dataset 1 (*N* = 11)	Dataset 2 (*N* = 13)
Female	9	6
Male	2	7
< 40 years	2	1
41 - 50 years	1	3
51 - 60 years	3	1
61 - 70 years	2	4
71 - 80 years	3	4
Spouse	9	6
Sister	0	2
Daughter/son	2	5
Bereaved caregiver	11	0
Current caregiver	0	13

### 2.1 Participants and data collection

While the primary studies included interviews with FCs, which were previously considered from both the patients’ and FCs’ perspectives, the current study aims to understand FCs’ experiences specifically. To provide context and outline the methodology used in the original data collection, we will briefly summarize the earlier primary studies.

#### 2.1.1 Dataset 1: *Silent voices: Family caregivers' narratives of involvement in palliative care* (*Tarberg et al., 2019*)

This study employed a qualitative design using a narrative approach (Holloway and Freshwater, 2007) to uncover FCs’ involvement in palliative care. The research question was as follows: How do FCs experience information and involvement in the different phases of palliative care? The study sample consisted of 11 bereaved FCs who had lost family members to cancer 3–12 months prior to data collection and who were selected through purposive sampling between November 2016 and May 2017 with the help of oncology nurses. Additional eligibility criteria included the FCs’ close involvement in their family members’ palliative care journey, which involved both primary and specialist healthcare, age over 18 years, and able to speak and read Norwegian. Interviews took place at the participants’ homes, except for two, who chose a community institution as the interview location. The first author conducted and transcribed the individual narrative interviews, which ranged from 50 to 180 min. In these interviews, the participants were given many opportunities to freely tell their own stories, including detailed accounts of how they had experienced palliative care. Their personal stories were centred, and the interview guideline questions were used only to direct the conversation. Table 2 presents examples of these questions.

**Table 2:** Examples of questions guiding the interviews.

	-Can you tell how you experienced the palliative care pathway?
Individual narrative interviews	-How did you experience being involved in the different phases of the palliative care pathway?
	-How did you experience the information you received in different phases of the palliative care pathway?

#### 2.1.2 Dataset 2: *Dignity of older home-dwelling women nearing end-of-life: Informal caregivers’ perception* (*Staats et al., 2021*)

Drawing on Gadamer’s philosophical hermeneutics (2010), this study investigated informal FC`s perspectives related to what contributing to promote or hinder dignity among older home-dwelling women with incurable cancer nearing their end of life. Thirteen FCs were selected through purposive sampling between November 2018 and December 2019, with the help of cancer co-ordinators. The interviews took place in the FCs’ homes, with two interviewees choosing a work location. Eligibility criteria included age over 18 years, providing informal end-of-life care for a woman aged over 65 years living with incurable cancer at home, and proficiency in speaking and reading Norwegian. The last author in the current study conducted individual in-depth interviews, which were guided by semi-structured, adaptable interview questions (Table 3). The interviews were all recorded, transcribed verbatim, and lasted between 50 and 81 min. The participants were encouraged to describe their experiences in their own words, including detailed accounts of their everyday caregiving practices, interactions with healthcare services, and situations they perceived as dignity-preserving or -degrading for their family members.

**Table 3:** Examples of questions guiding the interviews.

	Can you describe how its `like to be a family caregiver when caring for your wife/mother – and whether this affects your experience of dignity in your everyday life?
Individual in-depth interviews	Can you describe a dignity degrading situation?
	How do you think the municipal health service can contribute to a dignified death for your wife/mother/sister?

### 2.2 Secondary analyses using Gadamer’s hermeneutical circle

When employing Gadamer’s (2010) hermeneutical methodology, data interpretation occurs at multiple levels and is an iterative, circular process called the hermeneutical circle, which involves a continuous dialogue between the whole and its parts, each contributing to and enriching the understanding of the other (Malpas and Gander, 2015). When analyzing the data for the this paper, the first and last authors started the interpretive cycle by individually reading the clean, uncoded transcriptions from both primary studies. At the first level of interpretation, we conducted a detailed analysis of specific text segments. Before engaging the rest of the research team, we shared our reflections, questioned each other’s interpretations, and explored potential discrepancies across the two studies. In the second level of interpretation, we returned to the whole text with a more nuanced understanding of its parts and presented the preliminary emerging patterns to the rest of the research team. We then collectively navigated from the initial ideas and text segments to the overall interpretation of the text and back, which aligns with Gadamer’s (2010) hermeneutical methodology. Throughout this process, we paid close attention to our understanding as we identified and formulated themes and subthemes that represented our final interpretive understanding. The first and last authors reread all the interviews thoroughly and found that thematic saturation in relation to the research question was achieved. This was discussed and supported by all authors.

### 2.3 Preunderstandings

The research team’s preunderstandings were not detached or neutral from the subject under study. All authors are registered nurses with varying work and research experience with FCs and end-of-life care. We actively debated and questioned our preunderstandings to maintain transparency and trustworthiness (Lincoln and Guba, 1985). To ensure *credibility*, we consistently reflected on our diverse backgrounds and expectations to prevent premature conclusions related to the research questions during data interpretation. To establish *dependability*, we revisited and revised our initial findings multiple times, which encouraged us to explore various interpretive possibilities. To improve the *confirmability* of our data, we consciously attempted to divorce ourselves from prior preconceptions, ensuring that our findings were not influenced by personal biases. To provide *transferability*, we methodically illustrated the connection between the findings and the collected data and offered a detailed description of the context in which the findings were produced. Lastly, to enhance *authenticity*, we endeavoured to present the participants’ experiences and perceptions accurately and respectfully by quoting their words verbatim. In summary, considering that our preunderstandings could influence the interpretation of the empirical data, we actively scrutinized these preconceptions through critical reflections in our research meetings, which collectively contributed to the trustworthiness of our study.

### 2.4 Ethical considerations

In the primary studies, all participants were deemed vulnerable because they were either current (blinded) or bereaved FCs (blinded). As such, heightened ethical considerations and sensitivity were necessary to safeguard these FCs during the recruitment and data collection stages. For instance, the participants were given generous time to decide on their participation and respond during the interviews. Furthermore, the first and last authors used their oncology nursing backgrounds to demonstrate sensitivity and empathize with the challenges experienced by FCs. After the interviews, the FCs were offered follow-up contact with the municipal cancer nurse. Moreover, all participants received an informed consent letter with assurances of anonymity and confidentiality, which was completed prior to their inclusion in the study. In both primary studies, these letters also mentioned that the resulting data might be used in future publications. Both studies adhered to the principles of the Declaration of Helsinki (WMA Declaration of Helsinki, 1964) and received approval from the Norwegian Agency for Shared Services in Education and Research (reference numbers 2016/960–25 and 138,698).

## 3 Results

We identified three main themes related to FCs’ role in the home-based palliative care landscape positioned between healthcare professionals and patients: *being lost in an unclear palliative care pathway, feeling invisible in the caregiving role*, and *being assigned unclear responsibilities and tasks beyond capacity*.

### 3.1 Being lost in an unclear palliative care pathway

The FCs providing palliative care often described feeling like “pawns” who were expected to manage the practical aspects of their family members’ treatment without adequate support from healthcare professionals. Many expressed their overwhelming healthcare burden, which was encapsulated in sentiments such as “If I don’t fix it, no one fixes it” (Dataset 2, 41–50 years, daughter). Compounded by frequent turnover among healthcare professionals, the lack of predictability and planning left FCs feeling stranded and disoriented. One FC shared how this chaotic system made it difficult to know who to contact when they needed help:

You get completely exhausted from it. Because I don’t know who I should relate to. You don’t know who to call; you don’t know who to speak to. Because there are so many different people and names (D2, 41–50 years, daughter).

The absence of structured planning forced FCs to compensate for gaps in the provision of care, such as handling tasks typically managed by healthcare professionals. For instance, one FC described how they had to organize picking up a new nebulizer on Christmas Eve due to a lack of co-ordination between themselves and their family member’s healthcare providers: “I called home care nursing, but they couldn’t do it. So, I went on Christmas Eve, but then there were no medicines” (D2, 41–50 years, daughter).

The FCs perceived the palliative care pathway as treatment focused, with little room for their involvement. One participant described this as a “helpless” experience: “Everything is relegated to the healthcare system, to doctors – a totally different world” (D1, 61–70, wife).

With the minimal involvement of FCs in collaborative healthcare chains, communication within the healthcare system was often fragmented. FCs described their frustration when healthcare providers were unaware of each other’s decisions, further complicating their efforts to care for their family members. In contrast, oncology nurses were highlighted as pivotal in bridging communication gaps. FCs described these professionals as key intermediaries who explained how the healthcare system worked, provided practical insights, and recognized the FCs as individuals with needs of their own. This considerate support alleviated some of the FCs’ stress and gave them a clearer understanding of their roles in home-based palliative care, including upcoming events:

She, the oncology nurse, was exceptional. She told us, ‘Many decide to keep their loved ones at home, but it’s not always feasible, so take your time and figure it out.’ There was no pressure (D1, 71–80 years, wife).

Hence, the oncology nurse’s role in fostering clear communication and providing empathetic guidance was crucial in helping FCs navigate unclear palliative care pathways.

### 3.2 Feeling invisible in the caregiving role

Many FCs felt that they were not noticed or invisible despite their significant contribution to patient care. They often found that no one directly asked them about their well-being and needs. Some FCs acknowledged that the patient was the primary focus of healthcare professionals; however, others expressed difficulties regarding this lack of recognition, as they wanted to be involved in the care team supporting their family members:

This conversation has never been a topic at all. I know that my mother has received several invitations to a conversation concerning her well-being, but as a relative. No. Never received anything about it (D1, 31–40 years, son).

The FCs expressed struggling with their emotions, as they wanted to both be caregivers and support their family members; however, they experienced this as a daunting task. The FCs were aware that they would soon have a future alone without their terminally ill family member, while concurrently providing emotional support to them. This dichotomy in emotions and uncertainty underscores the complexities inherent in caregiving during the palliative phase: “You should both deal with the thoughts that his life is soon over and at the same time you must be a family caregiver who is supportive” (D1, 51–60 years, wife).

Uncertainty about their patients’ deterioration and caregiving trajectory further heightened the FCs’ stress, as they often felt unprepared for what lay ahead:

When she becomes very ill. What do I do? We have not talked about that. I dare not even discuss it. And when she can no longer walk on her own, things will become more difficult. I do not know how to solve that (D1, 31–40 years, husband).

The FCs were unsure whether it was their responsibility or that of healthcare professionals to initiate such conversations with their patients. While many FCs believed that the responsibility belonged to healthcare professionals, they doubted whether these discussions would ever take place. This lack of guidance left FCs feeling isolated and unprepared for the future. The interviewees also described the emotional toll of shifting roles within the family dynamic:

It’s as though I become the adult, and she becomes somewhat more the one who needs to be cared for. I feel that I must be the strong one … I couldn’t break down, even if I wanted to (D2, 41–50 years, son).

Coupled with high expectations from healthcare professionals, family members, and even themselves, this role reversal further complicated their caregiving experience. Consequently, the role of FCs gradually shifted from being a member of the family to solely caregivers.

### 3.3 Being assigned unclear responsibilities and tasks beyond capacity

The FCs often expressed feeling overwhelmed by the need to adopt responsibilities that should really be handled by healthcare professionals, which were then compounded by a lack of sufficient information or insufficient dialogue with healthcare professionals. This lack of communication left FCs uncertain about what to anticipate:

We were not considered in need of information. We had to request meetings with the doctor ourselves. We knew she was ill, or at least we thought so. The absence of information made us desperate (D1, 31–40 years, son).

Being alone in interpreting their patient’s condition without adequate support or competence caused significant feelings of unsafety and unpredictability among several FCs, who emphasized the need for clear communication and better dialogue to avoid misunderstandings:

When it comes to communication … We are humans, not just names in medical journals. It’s especially important to have a good dialogue with us as family caregivers so that misunderstandings can be avoided (D2, 61–70 years, husband).

The lack of clarity regarding responsibilities often led to FCs assuming unassigned tasks, such as arranging appointments, picking up medications, and managing their patients’ daily care needs. The FCs perceived this lack of recognition regarding their time and care efforts as an additional burden. While everything revolved around care for their family members, the FCs had to monitor numerous aspects of their care, such as providing nutrition, assisting with toilet visits, and potentially undesirable hygienic accidents. Many FCs often expressed feeling overwhelmed by the responsibilities they undertook for their patients, exacerbated by a lack of sufficient information and dialogue. One FC described how their patient’s deteriorating health intensified their responsibilities, with little support from healthcare professionals outside scheduled home care visits:

His health condition deteriorated significantly. I was very scared. He could hardly walk, and I had to help him and watch him, night and day. I told the home nurse, “I don’t know how long I can handle it” (D1, 61–70 years, wife).

For many FCs, this was their first experience caring for a seriously ill family member at home. They often did not know what to expect from healthcare services and considered the boundaries of responsibilities between themselves and healthcare professionals to be unclear: “I don’t know what my responsibility is. What can home care nursing personnel do and not do?” (D2, 51–60 years, daughter). These unclear boundaries of healthcare responsibilities between FCs and home care services led to confusion and the need for clarification. One FC described herself as “the contact person for everything that doesn’t work,” while others highlighted tasks that exceeded their competence, such as monitoring fluid accumulation, changing pain patches, or assessing the need for increased pain relief. This lack of clarity often created ambiguity in their relationships with healthcare professionals:

the fact that they are with us, that we are properly informed about what expectations they have … that we do not imagine that we should do more than told, not having wrong expectations that can lead to misunderstandings (D2, 51–60 years, daughter).

At times, FCs resisted assistance from home care services, either because they felt obligated to continue caregiving or struggled to let go of the responsibility: “If I had asked for help earlier … Because when he died, I was so worn out, I had nothing left to give” (D1, 51–60 years, wife).

The FCs also faced significant dilemmas, which healthcare professionals often presented as alternatives to care but could be particularly burdensome, especially when the FCs felt they lacked the competence to make informed choices, such as whether their patients should proceed with treatment or not. When their critically ill family member was cared for at home, their FCs found the responsibility particularly overwhelming:

We called the emergency services. They said it was not certain that he would tolerate the transport to the nursing home, and it was up to me whether he should go there or stay at home (D1, 61–70 years, wife).

While caring for their patients at home gave the FCs a sense of control, it also intensified their healthcare responsibilities. Many FCs suggested that healthcare professionals should be more attentive to signs of caregiver exhaustion, especially when continuity of care was provided by the same professionals, who could better understand their challenges. A clear preference emerged for more proactive involvement from healthcare services to ensure that the FCs were not left to navigate complex medical decisions alone, which would alleviate some of the overwhelming responsibilities and offer FCs relief and the support they need during demanding situations.

## 4 Discussion

This study explores the pivotal role of FCs in home-based palliative care and obtains a deeper understanding of their experiences and challenges as they navigate their often-undefined roles between healthcare professionals and patients. The findings reveal that FCs often feel lost in unclear healthcare pathways and struggle to feel recognized in their caregiving role while simultaneously trying to maintain their role as close family members. These FCs are frequently assigned healthcare tasks beyond their capacity, leading to ethical dilemmas and a sense of being passive participants in the healthcare system, which expects them to manage the practical aspects of treatments without adequate support from healthcare professionals. Fragmented and inconsistent communication within the healthcare system further disconnects FCs from key discussions, which makes it difficult to anticipate the next healthcare steps. These findings highlight the challenges faced by FCs in navigating the complex and demanding caregiving landscape, which we will discuss in relation to the literature and the theoretical framework of health literacy.

### 4.1 Navigating ambiguous roles in the palliative care pathway

In this study, FCs highlighted the challenges of navigating their roles within treatment-focused palliative care pathways. Policies such as the Norwegian National Health and Co-ordination Plan 2024–2027 (Ministry of Health and Care Services, 2024) emphasize the vital role of FCs and the importance of their involvement at all healthcare levels. However, the practical implementation of these policies often fails to meet expectations. While communication with home care services and general practitioners was maintained, FCs reported being excluded from healthcare discussions despite their central caregiving role, which left them feeling frustrated. Some FCs described feeling like pawns in a system that disregarded their crucial and indispensable knowledge of the patients, in addition to their perspectives of palliative care. These findings are aligned with Dillon et al. (2024) and the systematic review by Engel et al. (2023), who found that FCs often lack sufficient emotional and practical support, as well as recognition of their crucial roles from healthcare professionals and their social networks, which further exacerbates their burden. Additionally, this study found that FCs were often left to compensate for logistical inefficiencies, such as arranging transport, managing prescriptions, and handling treatment follow-ups, without assistance from healthcare professionals. This is consistent with Rakic et al. (2018), who highlighted how FCs felt unsure when providing healthcare for family members at home and were pressured by both patients’ and healthcare professionals’ expectations when combined with lack of support, often making these FCs’ caregiving roles seem invisible. Many interviewees observed that almost no one – neither healthcare professionals nor other family members – asked how they were coping, further contributing to their sense of isolation.

In Norway, a patient-centred approach known as “What matters to you” (Ministry of Health and Care Services, 2020a) is well recognized among healthcare professionals. The Norwegian Patient and User Rights Act acknowledges that FCs may experience a greater burden than patients but receive comparatively less support (Norwegian Ministery of Health and Care Service-Patients and User Rights, 1999). Despite these frameworks, national guidelines and legislation often lack nuance and follow up. Paradoxically, if FCs must legally have a more visible role, their patients must release healthcare professionals from their duty of confidentiality (Norwegian Directorate of Health, 2017), which creates an additional barrier to more effectively integrating FCs into healthcare processes. Nevertheless, health literacy emerges as a crucial factor in helping FCs to manage their caregiving roles and stand firm in their knowledge of their patients. As highlighted by Cianfrocca et al. (2018), it is crucial for healthcare professionals to empower and facilitate FCs by prioritizing health education so that they can effectively fulfil their roles, reduce their caregiving burden, and enhance overall well-being.

However, significant challenges persist in the practical implementation of health literacy initiatives. For FCs to have more defined roles as caregivers and to benefit from improved health literacy, it is essential that they engage in healthcare planning alongside healthcare professionals. In our study, this need was particularly evident when the turnover of healthcare professionals was high, leaving FCs feeling even more excluded from the Norwegian healthcare system, which was difficult to navigate and interpret. These findings are aligned with earlier studies (Matthys et al., 2022; McDonald et al., 2018; Naoki et al., 2018), which revealed that insufficient support for FCs often places a disproportionate burden on those delivering informal palliative care. Furthermore, low health literacy can intensify the challenges faced by FCs, contributing to heightened stress, social isolation, and mental health difficulties (Martínez-Santos et al., 2021). Therefore, it is important to make these challenges and targeted interventions visible to acknowledge FCs in their caregiving roles and to help them manage their own health better. The interviewees identified one such intervention, which involved the pivotal role of one group of healthcare professionals in alleviating their stress. Specifically, oncology nurses were highlighted as key intermediaries, as they offered practical insights, facilitated information sharing, and helped caregivers better understand their essential roles within the healthcare process. By fostering clearer communication and offering guidance, oncology nurses effectively helped FCs navigate the complexities of palliative care.

### 4.2 The weight of responsibility and decisions

In this study, the FCs often described themselves as passive participants in the healthcare system, which gave them significant practical and medical responsibilities without adequate support from healthcare professionals. This finding is aligned with previous research showing that FCs frequently feel overwhelmed by their caregiving burden and the numerous responsibilities they must manage, many of which carry unforeseen consequences (Rakic et al., 2018). Despite expressing their boundaries and needs, FCs often assumed more responsibilities than they felt capable of managing, largely due to inadequate healthcare support and indirect expectations from healthcare professionals (Eriksen et al., 2025).

In particular, medical and care responsibilities, such as pain management, medication monitoring, injections, assistance with toilet visits, and undesirable hygienic accidents, were commonly shifted to FCs. Although these responsibilities were rarely assigned to FCs, the routinely ended up taking them because the sensed, or inferred from the situation, that such involvement was expected of them. This absence of clear guidelines and professional support often forced FCs to make independent decisions without adequate knowledge or training, which in turn increased their distress. Providing FCs with practical guidance on pain control, recognizing signs of patient decline, and understanding the dying process are essential to equip them adequately for these tasks (Dose et al., 2015; Pop et al., 2022). However, studies consistently show that healthcare professionals often underestimated FCs’ need for information and direct explanations, particularly regarding their patients’ illnesses, prognoses, and end-of-life care (Røen et al., 2018; Collins et al., 2017). To address these challenges, health literacy emerges as a critical factor in enabling FCs to navigate their caregiving responsibilities because literate caregivers have the knowledge and competencies to interpret medical guidance, make informed decisions, and manage their complex caregiving tasks (Liu et al., 2020). Caregivers with higher levels of health literacy experience a lower caregiver burden and are better prepared to meet their family members’ healthcare needs (Zahedi et al., 2025). As Yuen et al. (2018) highlighted, however, FCs are not direct recipients of healthcare and often lack access to firsthand healthcare information, which is reserved for the patient. Challenges such as limited privacy during meetings with healthcare professionals, the lack of tailored information, and insufficient recognition of their caregiving role further complicate FCs’ ability to fulfil their responsibilities effectively. Although FCs have no formal obligation to follow up on their home-dwelling family members during serious illness, the Norwegian caregiver guidelines (Norwegian Directorate of Health, 2017) emphasize that municipal healthcare services must establish systems for caregiver involvement, which should include training, support, and guidance at multiple levels to ensure FCs receive the help they need. Nevertheless, there is a need to provide formal assistance in the home to improve support for FCs’ care of their family members.

Unclear boundaries between professional and caregiver responsibilities also affect the involvement of FCs in healthcare-related decision-making. In our study, the FCs reported feeling overwhelmed by the numerous healthcare decisions they had to make while simultaneously being excluded from key healthcare discussions, which left them uncertain about the next steps in their family members’ treatment. The absence of clear communication channels increased their stress, as FCs were left to navigate medical decisions without structured guidance. Earlier studies emphasized the importance of involving FCs in planning and treatment decisions while providing adequate social and emotional support, which ensures that FCs do not feel obligated to bear the sole responsibility of providing home-based palliative care (Adejoh et al., 2021; Woodman et al., 2016).

In our study, the FCs struggled to understand where their responsibilities ended, and professional care began. This overlap underscores the importance of health literacy in empowering FCs to navigate the healthcare system, interpret medical information, and make informed decisions (Yuen et al., 2024). While FCs do not hold formal responsibility for caregiving, they often experience significant pressure and a sense of duty to care for their sick family members. To prevent FCs from becoming overwhelmed, there is a pressing need for healthcare professionals to provide clear communication, accessible support systems, and respite services.

## 5 Conclusion

It is crucial for healthcare professionals to prioritize dialogue, effective communication, and consistent support to provide more formal assistance to FCs and their critically ill family members at home. FCs must navigate their ambiguous roles and cope with the decisions and responsibilities related to providing home-based palliative care. While their patients may maintain a sense of normalcy when receiving healthcare at home, FCs often experience a starkly different reality, which is marked by emotional and practical challenges.

Addressing the invisibility of FCs within the palliative care framework requires increasing their involvement in healthcare planning and fostering collaborations between FCs and healthcare professionals. Improved communication, structured emotional support, and formal recognition of their contributions are essential. Hence, it is necessary to establish a structured and proactive approach to integrating FCs into the healthcare system.

Healthcare professionals should focus on providing clear communication to ensure that FCs fully understand their roles and receive the guidance necessary to manage their caregiving responsibilities effectively. Transparent care pathways and stronger collaborations with FCs reduce uncertainties, alleviate emotional distress, and prevent them from being left to navigate complex medical decisions alone.

Although the role of FCs in the palliative care pathway is often undefined, it has one of the most significant impacts on palliative care because FCs are central to ensuring the well-being of patients at their end of life. Recognizing and supporting this role is vital to improving outcomes for both FCs and patients alike. However, it appears paradoxical that the legal requirement for patients to consent to waiving medical confidentiality may, in practice, hinder the involvement of FCs in home-based care. This highlights the critical importance of advanced health literacy among FCs, as competency enhances their capacity to contribute meaningfully to the healthcare process and to advocate for their family members’ best interests in a confident and informed manner.

## Acknowledgements

We are grateful for the family caregivers’ trusting engagement in sharing their perceptions.

We would like to thank Helse Møre and Romsdal hospital trust and the University of Bergen for sharing data with us.

## CRediT authorship contribution statement

**Anett Skorpen Tarberg:** Writing – review & editing, Writing – original draft, Validation, Methodology, Formal analysis, Data curation, Conceptualization. **Bodil Aarmo Brenne:** Writing – review & editing, Writing – original draft, Validation, Methodology, Formal analysis, Conceptualization. **Kristin Jeppestøl:** Writing – review & editing, Writing – original draft, Validation, Methodology, Formal analysis, Conceptualization. **Bente Egge Søvde:** Writing – review & editing, Writing – original draft, Methodology, Formal analysis, Conceptualization. **Katrine Staats:** Writing – review & editing, Writing – original draft, Validation, Methodology, Formal analysis, Data curation, Conceptualization.

## Declaration of competing interest

The authors declare the following financial interests/personal relationships which may be considered as potential competing interests:

Anett Skorpen Tarberg reports administrative support and article publishing charges were provided by Norwegian University of Science and Technology. If there are other authors, they declare that they have no known competing financial interests or personal relationships that could have appeared to influence the work reported in this paper.

## Data Availability

The data will be made available upon request to the corresponding and last author. Please note that the data is provided exclusively in Norwegian.
